# Test-retest reliability for common tasks in vision science

**DOI:** 10.1167/jov.22.8.18

**Published:** 2022-07-29

**Authors:** Kait Clark, Kayley Birch-Hurst, Charlotte R. Pennington, Austin C. P. Petrie, Joshua T. Lee, Craig Hedge

**Affiliations:** 1 University of the West of England, Department of Social Sciences, Bristol, UK; 2 Aston University, School of Psychology, College of Health & Life Sciences, Birmingham, UK; 3 Cardiff University, School of Psychology, Cardiff, UK; 4 University of Sussex, School of Psychology, Sussex, UK

**Keywords:** individual differences, perception, attention, visual cognition

## Abstract

Research in perception and attention has typically sought to evaluate cognitive mechanisms according to the average response to a manipulation. Recently, there has been a shift toward appreciating the value of individual differences and the insight gained by exploring the impacts of between-participant variation on human cognition. However, a recent study suggests that many robust, well-established cognitive control tasks suffer from surprisingly low levels of test-retest reliability (Hedge, Powell, & Sumner, 2018b). We tested a large sample of undergraduate students (*n* = 160) in two sessions (separated by 1–3 weeks) on four commonly used tasks in vision science. We implemented measures that spanned a range of perceptual and attentional processes, including motion coherence (MoCo), useful field of view (UFOV), multiple-object tracking (MOT), and visual working memory (VWM). Intraclass correlations ranged from good to poor, suggesting that some task measures are more suitable for assessing individual differences than others. VWM capacity (intraclass correlation coefficient [ICC] = 0.77), MoCo threshold (ICC = 0.60), UFOV middle accuracy (ICC = 0.60), and UFOV outer accuracy (ICC = 0.74) showed good-to-excellent reliability. Other measures, namely the maximum number of items tracked in MOT (ICC = 0.41) and UFOV number accuracy (ICC = 0.48), showed moderate reliability; the MOT threshold (ICC = 0.36) and UFOV inner accuracy (ICC = 0.30) showed poor reliability. In this paper, we present these results alongside a summary of reliabilities estimated previously for other vision science tasks. We then offer useful recommendations for evaluating test-retest reliability when considering a task for use in evaluating individual differences.

## Introduction

Historically, vision scientists have assumed most human visual systems to be interchangeable. Perception and attention tasks have aimed typically to characterize a “standard observer” (e.g. Judd, 1933) and yield insight into the “average” cognitive and/or neural processes involved in human vision. Individual human participants have been merely a means to measure what are assumed to be fixed effects across the population, with many studies relying upon small sample sizes, especially in studies involving low-level psychophysics (Anderson & Vingrys, 2001). Indeed, many effects in perception and attention are so robust that the difference between two or more conditions is readily observed within almost any human participant (e.g. the Stroop effect; Haaf & Rouder, 2019; Stroop, 1935). Thus, the focus has been to characterize the human visual system as a standard system across the population and to minimize between-participant differences (often considered “noise”) rather than to consider the degree to which certain effects are seen in one individual versus another.

Although fixed effects across a population remain a central focus in vision science, over the past several decades, researchers have begun to explore systematic differences between individuals. This shift in focus toward understanding individual differences in vision can yield further insight into what systematic variance can tell us about the processes underlying perception and attention and how individuals’ characteristics interact with their visual systems (for reviews, see Mollon, Bosten, Peterzell, & Webster, 2017; Peterzell, 2016; Wilmer, 2008). Individual differences in vision have been identified according to variations in characteristics ranging from age (e.g. Roberts & Allen, 2016) to personality (e.g. Kaspar & König, 2012) to clinical disorders (Simmons et al., 2009) to intelligence and attention control (Tsukahara, Harrison, Draheim, Martin, & Engle, 2020). Likewise, individual differences in vision have been revealed across the spectrum of vision science study, from eye movements (e.g. Bargary, Bosten, Goodbourn, Lawrance-Owen, Hogg, & Mollon, 2017) and low-level sensory and motion processing (e.g. Golomb, McDavitt, Ruf, Chen, Saricicek, Maloney, Hu, Chun, & Bhagwagar, 2009) to higher-level processing in terms of both local versus global perception (e.g. de-Wit & Wagemans, 2016) and visual search (e.g. Biggs, Clark, & Mitroff, 2017).

In addition to assessing the relationship between performance on cognitive tasks and individual differences such as personality traits, some research has also evaluated the relationship between performance on the tasks themselves. In intelligence research, there is a theorized “common factor” (Spearman's *g*; Jensen, 1998) underlying performance on various intelligence tasks, with measures such as verbal and spatial intelligence sharing a substantial proportion of their variability (e.g. Johnson, Nijenhuis, & Bouchard, 2008). The same does not follow for basic visual tasks, with few relationships across tasks, such as visual acuity and Vernier discrimination (Cappe, Clarke, Mohr, & Herzog, 2014). However, there are a wide range of perceptual faculties, and factor analysis has revealed a general “attention” factor underlying some higher-level tasks, such as conjunction search and change blindness, but uncorrelated with others (e.g. attentional capture and inhibition of return; Huang, Mo, & Li, 2012). This general “attention” factor may also be more finely grained with separate clusters representing similar performance within individuals on attentional faculties, such as spatiotemporal attention versus sustained attention (Skogsberg, Grabowecky, Wilt, Revelle, Iordanescu, & Suzuki, 2015); subcomponents of attention such as these can capture individual differences in performance in both traditional experimental and neuropsychological paradigms (Treviño, Zhu, Lu, Scheuer, Passell, Huang, Germine, & Horowitz, 2021). There is much to be learned about perceptual processes through the study of individual differences; however, in order to do so effectively, we must assess whether the tasks we are using are suitable for the measurement of individual differences.

The fact that a well-established, replicable task may be appropriate for producing consistent within-participant effects while being unsuitable for examining individual differences between participants may seem counterintuitive (Hedge et al., 2018b). In some respects, vision scientists believe their work to be so robust as to be immune to the issues of reproducibility that have plagued other fields within psychology (e.g. Holcombe, Ludowici, & Haroz, 2019). Our effects are generally quite replicable (Zwaan, Pecher, Paolacci, Bouwmeester, Verkoeijen, Dijkstra, & Zeelenberg, 2018), and we tend to think that our measures are telling us something meaningful about what is happening perceptually, cognitively, and/or neurally. In this paper, we are not arguing that the tasks do not capture the mechanisms that they are intended to; rather, we are aiming to raise awareness that the same tasks that are effective at identifying the “standard observer” are not necessarily appropriate for the assessment of individual differences in those mechanisms.

To understand why our measures may be potentially unsuitable for the investigation of differences *between* participants, we must first consider why they are so good at telling us about consistencies across a population. Recalling the origins of cognitive science, the aim was to understand the human mind (i.e. “all minds”) as a computer – receiving input and producing output according to an existing neural architecture that is common across the species. When our goal is to identify the “standard observer,” then the sample average is the signal we are interested in, and variability across observers is noise. In contrast, variability across observers is our signal of interest in the study of individual differences, and measurement error is the noise (c.f. Novick, 1966). Measurement error consists of factors, such as trial-to-trial variability, in an observer's performance, as well as “state” factors that are known to fluctuate over time, such as mood (e.g. Booth, Schinka, Brown, Mortimer, & Borenstein, 2006), sleep quality (e.g. Nebes, Buysse, Halligan, Houck, & Monk, 2009), and phase of the menstrual cycle (Farage, Osborn, & MacLean, 2008). The key point is that where the researcher tries to maximize between-participant variance in the study of individual differences, this is a nuisance to the researcher interested in the average observer (Cronbach, 1957).

These different goals present a tension similar to one recently described as “the reliability paradox” (Hedge et al., 2018b). Hedge et al. (2018b) proposed that tasks that became popular for their robust within-subject effects (e.g. Stroop and Eriksen flanker) may have undergone selective pressures to minimize individual differences. For example, the classic Stroop (1935) task is widely used, and the effect has been consistently replicated in the literature (Ebersole et al., 2016; MacLeod, 1991). For a task to consistently produce a statistically significant effect at the group level, it is beneficial when between-participant variance is small. For the same average effect (e.g. 30 ms), a task with more variability will have a smaller effect size (Cohen's d_z_ = mean effect/standard deviation) and subsequently lower statistical power for a given sample size. When we then turn to individual differences, this same low level of variability will limit the reliability of the Stroop effect and the extent to which certain characteristics predict how susceptible one may be to it. This “paradox” reveals a discrepancy between tasks which are good for global reproducibility across a population relative to those that are good for individual differences research.

Many of the tasks examined by Hedge et al. (2018b) rely on the difference between two highly correlated condition scores (e.g. incongruent reaction time [RT] and congruent RT) as the key performance indicator, and there is a long history of reliability concerns associated with these difference scores (Cronbach & Furby, 1970; Lord, 1956). In contrast, many paradigms used by vision scientists do not rely on difference scores. For example, paradigms like multiple object tracking may take a mean or maximum number of objects tracked (Hulleman, 2005), and visual working memory (VWM) tasks typically use an equation to extract a measure of capacity (Cowan, 2001; Pashler, 1988). Nevertheless, a historical focus on the average observer and small samples may have optimized vision tasks for consistency across observers at the expense of their ability to reveal relationships with other individual differences (e.g. age, personality, and clinical symptoms). For example, it is often noted that estimates of working memory capacity typically range from three to five items in healthy adults (Cowan, 2001; Cowan, 2010). If performance across individuals is too similar, then our signal for individual differences is weak, and we will have difficulty separating it from measurement noise.

As interest in individual differences in attention and perception has increased, several recent papers have proposed a variety of pitfalls and criteria for sound research (e.g. Mollon et al., 2017; Wilmer, 2017), suggesting the relevance of test-retest reliability amongst their advice. Some recent studies have begun to include a retesting session in their protocol, but this is still not common practice, with many studies simply testing all participants on a given task once, administering a questionnaire, and then correlating the results. In the current study, we examined the test-retest reliability of four commonly used tasks in vision science. We selected a sample of tasks that spanned a range of perceptual and attentional processes and for which there were not test-retest reliability statistics already established in the literature (at the time of data collection). Specifically, we included a motion perception task (motion coherence), a peripheral processing task (useful field of view), a sustained attention task (multiple-object tracking), and a VWM task (change detection). We tested a large sample of participants across two sessions and assessed test-retest reliability for multiple dependent variables within each task. We present our results alongside previously reported test-retest reliability statistics for a wide range of additional tasks within vision science. We aim to promote consideration of test-retest reliability when exploring individual differences, yield a useful collection of the currently known test-retest reliability scores for vision science tasks, and provide recommendations for testing procedures and methodological considerations when investigating reliability.

## Methods

### Participants

One-hundred sixty psychology undergraduates (137 women, 2 unspecified, *M*_AGE_ = 20.73, SD = 3.96) from a UK university participated in the study across two testing sessions. All participants reported normal or corrected-to-normal vision. Participants provided informed consent, and the recruitment and testing protocol were approved by the Faculty Research Ethics Committee and adhere to the Declaration of Helsinki.

Our data were collected in conjunction with another project examining measures of social cognition (Pennington, Shaw, Ploszajski, Mistry, NgOmbe, & Back, in preparation), which aimed to recruit a minimum of 200 participants. To determine whether this sample size would be appropriate for our purposes, we conducted a precision estimate analysis, as a traditional power analysis has limited value for a reliability study. Ideally, we want our reliability to be as close to one as possible, rather than simply, rejecting the null hypothesis, which is typically that the correlation is zero. In practice, we want to know how precise our estimates of the reliability are for a given sample size. We adapted the approach of Doros and Lew (2010) to evaluate this. Based on these analyses, we aimed for a target sample size of 150.

Ultimately, we collected data from 160 participants due to ongoing sign ups before participation was closed. We then updated the precision estimate accordingly. For 160 participants, we evaluated the expected (average) 95% confidence interval (CI) for a meaningful range of correlations (see Table 1). We simulated two correlated variables in a population of 100,000 individuals. We did this for three different correlation values, given in the “True r” column. We then took 10,000 random samples of 160 of these individuals and report the average intraclass correlation coefficient (ICC) and 95% CI. The key point is that the CIs are narrow. A wide CI would mean that our data were consistent with a wide range of correlations, which would be of limited value in determining whether a task was reliable enough to be used.

**Table 1.** Average intraclass correlation coefficient (ICC) and 95% CI observed in simulated data.

True r	ICC [95% CI]
0.4	0.4 [0.26, 52]
0.6	0.6 [0.49, 69]
0.8	0.8 [0.73, 85]

*Note*. The “True r” refers to a correlation imposed in the simulated data.

### Design and procedure

Participants completed four vision tasks that were administered as the second group of tasks in conjunction with a related project looking at test-retest reliability for social cognition tasks (Pennington et al., in preparation). Participants completed the social and vision tasks in two 2-hour sessions taking place one to three weeks apart. All participants completed each task in the same order, and the order of the tasks were the same for both sessions 1 and 2. We kept these consistent to avoid potential participant × order interactions from influencing our results.

Up to four participants were tested at the same time at a multitesting station separated by dividers. Participants were seated at a station in a dimly lit room, and stimuli were presented at a viewing distance of 57 cm (except for useful field of view [UFOV], which was presented at a viewing distance of 30 cm) with head restraint. All participants were instructed to complete each task by an experimenter who was present throughout the session to ensure compliance. To minimize fatigue, participants were given frequent breaks throughout testing. PsychoPy software (Peirce, Gray, Simpson, MacAskill, Höchenberger, Sogo, Kastman, & Lindeløv, 2019) was used to present stimuli and collect responses for all tasks. All experimental materials, code, raw data, and analyses for each behavioral task are available via the Open Science Framework (https://osf.io/gtusw/).

#### Useful field of view

**Figure 1.**
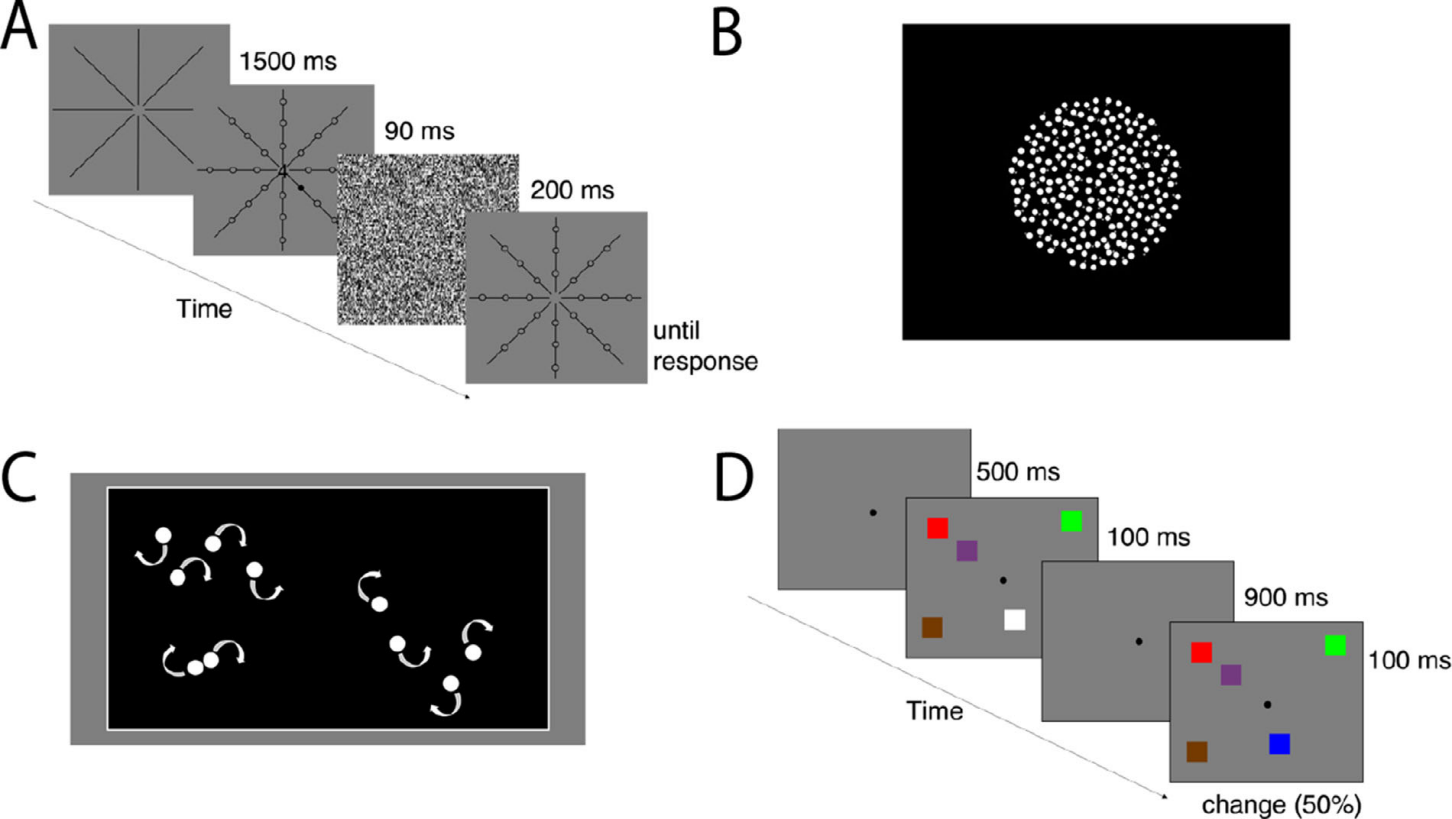
Example trials of the behavioral tasks. *Note*. (**A**) An example trial of the UFOV task with the number four as the central target, and a peripheral target 7.07 degrees from fixation among 23 distractors. (**B**) An illustration of the random dot stimulus patch. (**C**) An example trial of the MOT task with five target circles among five distractor circles. (**D**) An example trial of the VWM task depicting a five-item change trial.

Each participant was assessed using a modification of the UFOV paradigm (Ball, Beard, Roenker, Miller, & Griggs, 1988; Edwards, Vance, Wadley, Cissell, Roenker, & Ball, 2005). Before starting the task, participants were told that accuracy rather than speed was important. Each trial consisted of four successive displays (see Figure 1A). The first display consisted of eight cardinal/intercardinal arms, presented on a grey background (1500 ms), which radiated from a central 3 degrees fixation circle. The second display was presented for 90 ms and required identification of a central target along with localization of a simultaneous peripheral target. On each of the eight arms, three unfilled 1 degree diameter circles with a black outline were assigned to evenly spaced positions, resulting in 24 eccentricity locations: 10 degrees, 20 degrees, and 30 degrees from fixation on the cardinal arms, and 7.07 degrees, 14.14 degrees, and 21.21 degrees from fixation on the intercardinal arms. On each trial, one of the circles filled black (target), whereas the remaining 23 circles remained unfilled (distractors). Simultaneously, a number from one to nine was presented at the center of the array. Stimulus presentation was followed by a randomly generated grayscale dot array for 200 ms to prevent any residual afterimages on the screen. At test, the eight arms reappeared, and participants indicated the location of the target circle with a mouse click and typed which number appeared in the center of the array. The eight locations in the inner, middle, and outer eccentricities were repeated randomly five times resulting in 40 trials for each eccentricity and a total of 120 experimental trials overall. The proportion of trials for which participants responded correctly to the central target and location of the peripheral target within the inner, middle, and outer eccentricities were the primary dependent measures.

#### Motion coherence

Participants were shown random-dot kinematograms (RDKs; Appelbaum, Schroeder, Cain, & Mitroff, 2011; Newsome & Paré, 1988; Snowden & Kavanagh, 2006) consisting of high luminance white dots moving against a black background (see Figure 1B). Each array included 400 dots sized 7.5 pixels presented within a circular arena (9.0 degrees × 9.0 degrees of visual angle) for 440 ms. On any given trial, a proportion of the dots moved coherently to the left or right (signal dots), and the remaining dots (noise dots) moved in angular directions selected at random. Throughout the display sequence, the choice of which dots are signal and which are noise was randomized on each frame (Scase, Braddick, & Raymond, 1996). To prevent detection of motion by following the movement of a single dot, each dot had a fixed lifetime of 12 frames, after which it would disappear before being regenerated at a random place within the stimulus patch. The motion coherence (MoCo) began at 0.24; for example, for the first trial, the stimulus patch had 24% of the dots moving in a single direction (signal dots), either left or right, with the other 76% (noise dots) moving randomly. Participants indicated whether the dots moved coherently to the “left” or “right” by using the left and right arrow keys. The proportion of coherently moving dots was controlled and varied to the participant's detection threshold by a two-up, one-down staircase procedure. The motion coherence was reduced by 1% following a correct response and increased by 2% following an incorrect response. This staircase procedure estimates the proportion of dots that must move coherently for each participant to produce an 82% accuracy rate. Each participant received eight practice trials before threshold measurement began and received feedback about each response. This was followed by 105 experimental trials in total, 35 for every staircase; this number exceeds the number of trials typically used with similar tasks (e.g. 64 trials; Snowden & Kavanagh, 2006). The ratio of “signal” to “noise” dots required to determine the coherent motion direction (i.e. the motion coherence threshold) was the primary dependent measure. Individual threshold values were computed as the average of the three staircases.

#### Multiple-object tracking

Participants were asked to keep track of the locations of a subset of moving target circles among a field of identical randomly moving distractor circles. The display consisted of white circles (0.8 degrees × 0.8 degrees of visual angle) within a black box surrounded by a white border and a grey background (see Figure 1C). A randomly chosen subset of target circles “flashed” for 2 seconds (i.e. filled in black 4 times), then all the circles moved pseudo-randomly and independently for 6.5 seconds at a speed of 2.5 degrees/s. The number of distractors was always equal to the number of targets (e.g. 5 target circles among 5 distractor circles with a total of 10 circles in the array), and the same speed and duration was maintained across all experimental trials. When a circle reached a border, its motion was reflected off that border. Participants kept track of the locations of the circles that flashed, and when the motion stopped, they used a mouse to click on each target. Circles turned grey upon mouse-click and remained on the screen until the number of targets for that given trial was met. Five practice trials were completed followed by a total of 20 experimental trials. Three target items were presented on the first trial, and the number of target items (and corresponding number of distractors) was adjusted on each trial, using a one-up, one-down staircase procedure. If all target items were identified correctly, the number of targets was increased on the next trial; if one or more items was identified incorrectly, the number of targets was decreased on the next trial. The primary dependent measures were the maximum number of items tracked (i.e. the highest tracking load reached on the staircase) and the participant's threshold as defined by the mean number of items presented on the final four steps.

#### Visual working memory

VWM capacity was assessed using a Change Detection Paradigm (Luck & Vogel, 1997) where the stimulus array was composed of colored squares (1 degree × 1 degree of visual angle). We used eight colors: black (red, green, and blue [RGB] values = 0, 0, and 0), blue (0, 0, and 255), green (0, 255, and 0), red (255, 0, and 0), brown (116, 58, and 0), purple (116, 58, and 128), yellow (255, 255, and 0), and white (255, 255, and 255). Each array was comprised of two to seven squares displaced to the left and right of a central fixation circle on a grey background. The sample stimuli were presented for 100 ms, followed by a 900 ms retention interval. At test, the array briefly reappeared (100 ms) and was either identical or different to the sample array (see Figure 1D). The color of one item in the test array was different from the corresponding item in the sample array on 50% of the trials. Participants indicated whether there was a “change” or “no change” to the display using the “c” and “n” keys, respectively. One hundred eighty-four experimental trials were completed in total, with the opportunity for a break every 25 trials. To estimate VWM capacity, change detection accuracy was transformed into a *K* estimate using Pashler's (1988) formula: N × (H – FA)/(1 – FA), where *K* corresponds to the number of items maintained in VWM, N represents the set size, H is the hit rate (proportion of correct responses to change trials), and FA is the false alarm rate (proportion of incorrect responses to no-change trials). Estimates for VWM capacity were initially calculated for each set size; we then averaged these values across set sizes three to seven (omitting set size 2, where performance was near ceiling) to produce overall estimates of individual VWM capacity.

### Data analysis

Overall, 142 participants (88.75%) returned for session 2. If participants were missing data from either session 1 or 2, their data were not included in the analyses (MoCo = 19, multiple-objects tracking [MOT] = 18, UFOV = 19, and VWM = 18). In addition, four participants were excluded from the UFOV analyses because of not completing a full set of trials (*N* = 1) or poor performance on the number task (as defined by 5 standard deviations below the group mean in either session, *N* = 3). Participants whose average capacity values were negative in the VWM task were also excluded (*N* = 6); a negative capacity estimate is observed if the false alarm rate exceeds the hit rate and have no interpretable meaning in the analysis of VWM capacity (Morey, 2011). After these exclusions, 141 participants remained in the MoCo task, 137 in the UFOV task, 142 in the MOT task, and 137 in the VWM task.

In line with Koo and Li (2016), reliabilities were calculated using ICC using a two-way mixed-effects model for absolute agreement; all values were computed using MATLAB (MathWorks, 2020). The ICC takes the form:

$$\;\;\;\;\;\;\;\;\;\;\;\;\;\mathrm{ICC}\;=\;\frac{\mathrm{Variance}\;\mathrm{between}\;\mathrm{individuals}}{\begin{array}{c} \mathrm{Variance}\;\mathrm{between}\;\mathrm{individuals}+\mathrm{Error}\;\mathrm{variance}\\ \;\;+\;\mathrm{Variance}\;\mathrm{between}\;\mathrm{sessions} \end{array}}.$$

We also report the standard error of measurement (SEM) for each measure, as both measurement error and between-participant variation are important for interpretation of reliability. The SEM is the square root of the error variance term in the ICC calculation and reflects the 95% CI around an individual's observed score.

## Results

### Task performance

Descriptive statistics for each key measure for sessions 1 and 2 are summarized in Table 2. Participants’ MoCo thresholds were comparable to previous reports in the literature (e.g. Holten & MacNeilage, 2018). In the UFOV task, accuracy scores decreased as target eccentricity increased and accuracy scores for the center target were close to ceiling, as would be expected for young adults (Ball et al., 1988). For MOT, the maximum number of items that participants could track was marginally higher than demonstrated in previous studies (e.g. Trick, Hollinsworth, & Brodeur, 2009); however, this discrepancy may be due differences in the parameters employed. Although our K values for estimating VWM capacity are slightly smaller than reported by previous work (e.g. Dai, Li, Gan, & Du, 2019), it is important to note that our K values have been averaged across set sizes.

**Table 2.** Means (SD) for measures of the motion coherence (MoCo), useful field of view (UFOV), multiple-object tracking (MOT), and visual working memory (VWM) tasks.

Task	Measure	Session 1	Session 2
MoCo	Threshold (% coherent)	0.28 (0.10)	0.28 (0.10)
UFOV	Number accuracy	0.94 (0.06)	0.96 (0.05)
	Inner accuracy	0.79 (0.19)	0.91 (0.16)
	Middle accuracy	0.24 (0.17)	0.30 (0.20)
	Outer accuracy	0.13 (0.12)	0.15 (0.15)
MOT	Max items	5.93 (0.66)	5.94 (0.62)
	Threshold (number of items)	4.48 (0.67)	4.47 (0.80)
VWM	Capacity/K	2.34 (0.75)	2.39 (0.83)

### Task reliabilities

Typical interpretations of ICC values are: excellent (>0.80), good (0.60–0.80), moderate (0.40–0.60), and poor (<0.40) levels of reliability (Cicchetti & Sparrow, 1981; Fleiss, 1981; Hedge et al., 2018b; Landis & Koch, 1977). In the current study the VWM capacity measure showed good reliability. The motion coherence threshold reached a standard of good/moderate reliability. Within the UFOV task some measures showed better levels of reliability than others; the outer and middle accuracy measures reached a standard of good/moderate reliability, whereas the number accuracy measure reached a standard of moderate reliability, and the inner accuracy measure reached a standard of poor reliability. Overall, the MOT showed the lowest levels of reliability with the maximum number of items tracked reaching a moderate standard and the item threshold reaching a poor standard. All task reliabilities are summarized in Table 3. We also report Spearman's Rho correlation coefficients as an alternative approach to estimating reliability. As shown in Table 3, both approaches give similar reliability estimates.

**Table 3.** Intraclass correlations (ICC) and Spearman's Rho correlation estimates for the motion coherence (MoCo), useful field of view (UFOV), multiple-object tracking (MOT), and visual working memory (VWM) tasks.

Task	Measure	ICC [95% CI]	Rho [95% CI]
MoCo	Threshold	0.60 [0.48, 0.69]	0.57 [0.43, 0.68]
UFOV	Number accuracy	0.48 [0.33, 0.60]	0.47 [0.31, 0.61]
	Inner accuracy	0.35 [0.10, 0.54]	0.50 [0.36, 0.62]
	Middle accuracy	0.60 [0.44, 0.72]	0.65 [0.51, 0.75]
	Outer accuracy	0.74 [0.66, 0.81]	0.75 [0.63, 0.82]
MOT	Max items	0.41 [0.26, 0.53]	0.36 [0.20, 0.51]
	Threshold (number of items)	0.36 [0.20, 0.49]	0.31 [0.15, 0.45]
VWM	Capacity/K	0.77 [0.69, 0.83]	0.78 [0.73, 0.84]

**Figure 2.**
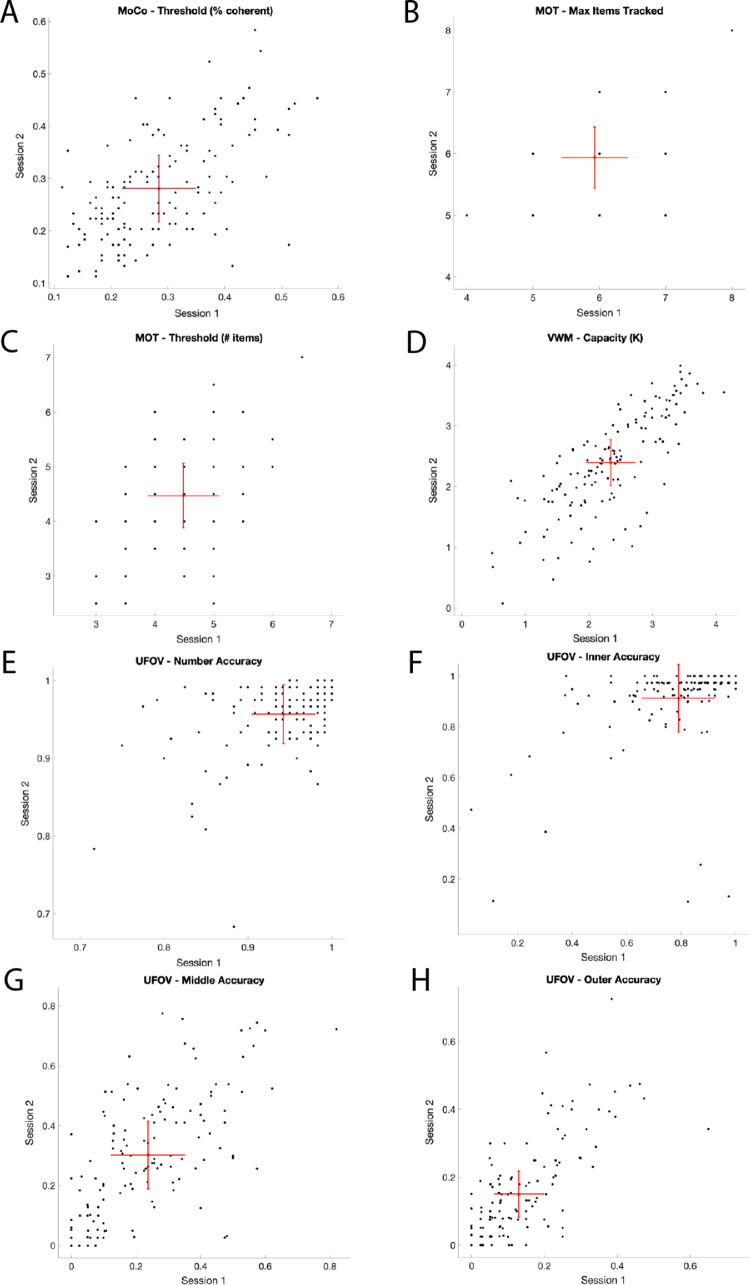
Reliability of the key measures from the motion coherence (**A**), multiple-object tracking (**B, C**), useful visual working memory change detection (**D**), and useful field of view (**E, F, G, H**) tasks. *Note*. Red markers indicate mean group performance from sessions 1 and 2. Error bars show ± standard error of measurement (SEM). Black markers indicate individual participant scores for session 1 and session 2; where multiple participants have the same score, black markers overlap.

The SEM for measures for each task are shown in scatterplots in Figure 2.

**Figure 3.**
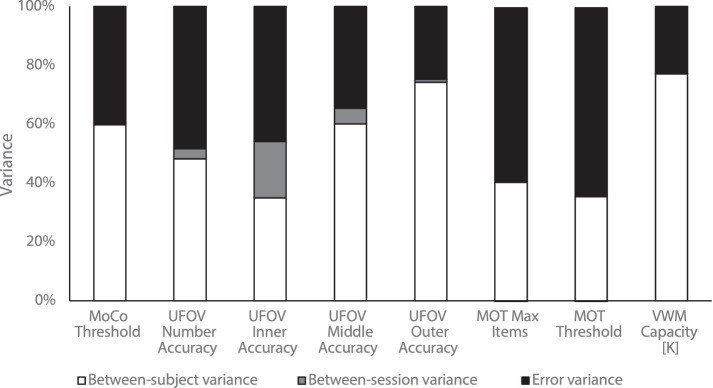
Variance components of the ICC for each behavioral measure. *Note*. The relative size of the variance components for each measure reported. The bar sizes are normalized according to the total variance for the measure and subdivided by variance accounted for by differences between participants (white), differences between sessions (grey), and error variance (black).

The relationship between reliability and the three components of variance used to calculate the ICC are shown in Figure 3 for each of the key measures. Task measures with higher ICC scores also showed higher between-participant variance. For example, between-participant variance is a relatively larger component of total variance for VWM compared to UFOV.

### Effects related to gender and practice

To assess potential effects of gender and practice, we conducted 2 (session) × 2 (gender) mixed ANOVAs of each measure. There were no significant interactions between session and gender. Men performed significantly better than women on two of the eight measures: UFOV outer accuracy, *F*(1, 135) = 7.29, *η^2^_p_* = 0.05, *p* = 0.008, and MOT threshold, *F*(1, 140) = 8.98, *η^2^_p_* = 0.06, *p* = 0.003; there were no significant gender differences on the other six measures. Levene's tests revealed unequal variances between men and women on session 2 for MOT mean items, *F*(1, 140) = 6.22, *p* = 0.01. Despite these differences in performance and variance, the ICCs for these measures were roughly equivalent between men and women (see Supplementary Materials A).

Significant practice effects were evident across all four of the UFOV measures (UFOV number accuracy: *F*(1, 135) = 6.25, *η^2^_p_* = 0.04, *p* = 0.01, UFOV inner accuracy: *F*(1, 135) = 21.48, *η^2^_p_* = 0.14, *p* < 0.001, UFOV middle accuracy: *F*(1, 135) = 10.65, *η^2^_p_* = 0.07, *p* = 0.001, and UFOV outer accuracy: *F*(1, 135) = 9.54, *η^2^_p_* = 0.07, *p* = 0.002) but not for the other four measures.

### Test-retest reliability from the vision science literature

Some previous studies have assessed test-retest reliability studies on common tasks in vision science. Table 4 summarizes test-retest reliabilities from previous studies, which include commonly used measures of attentional control, sustained attention, selective attention, and VWM.

**Table 4.** Summary of test-retest reliability from the literature.

Task	Measure	Study	Reliability	Correlation coefficient
Eriksen flanker	RT cost	van Leeuwen et al. (2007) E1	0.48	Pearson's *r*
		van Leeuwen et al. (2007) E2	0.48	Pearson's *r*
		Wöstmann et al. (2013)	0.91	ICC
		Paap & Sawi (2016)	0.52	Pearson's *r*
		Hedge et al. (2018b)	0.57	ICC
	Error cost	van Leeuwen et al. (2007) E1	0.29	Pearson's *r*
		van Leeuwen et al. (2007) E2	0.14	Pearson's *r*
		Wöstmann et al. (2013)	0.65	ICC
		Hedge et al. (2018b)	0.72	ICC
				
Posner cueing task	Cueing effect	Hedge et al. (2018b)	0.70	ICC
				
Navon task	Local RT cost	Hedge et al. (2018b)	0.14	ICC
	Local error cost	Hedge et al. (2018b)	0.82	ICC
	Global RT cost	Hedge et al. (2018b)	0	ICC
	Global error cost	Hedge et al. (2018b)	0.17	ICC
				
Digit vigilance test (DVT)	Task duration	Lee, Li, Liu, & Hsieh (2011)	0.83	ICC
				
Continuous performance task (CPT)	Commission errors	Weafer, Baggott, & de Wit (2013)	0.73	Pearson's *r*
		Wöstmann et al. (2013)	0.51	ICC
		Soreni, Crosbie, Ickowicz, & Schachar (2009)	0.72	ICC
	Omission errors	Weafer et al. (2013)	0.42	Pearson's *r*
				
Tasi test	% Hits	Fernández-Marcos, de la Fuente, & Santacreu (2018)	0.15	ICC
	% Commission errors	Fernández-Marcos et al. (2018)	0.23	ICC
	Mean RT	Fernández-Marcos et al. (2018)	0.85	ICC
				
Conjunctive Continuous Performance Task-Visual (CCPT-V)	Mean RT/hits	Shalev, Ben-Simon, Mevorach, Cohen, & Tsal (2011)	0.76	Pearson's *r*
				
Trees Simple Visual Discrimination (DiViSA)	Commission errors	Fernández-Marcos et al. (2018)	0.75	ICC
	Test duration (seconds)	Fernández-Marcos et al. (2018)	0.72	ICC
				
Conjunctive Visual Search Test (CVST)	Mean RT	Shalev et al. (2011)	0.52	Pearson's *r*
				
Adaptative Choice Visual Search (ACVS)	Optimal choice (%)	Irons & Leber (2018)	0.83	Pearson's *r*
	Switch rate (%)	Irons & Leber (2018)	0.77	Pearson's *r*
				
Mouse Click Foraging Task (MCFT)	Mean run length (feature condition)	Clarke et al. (2022)*	0.70	Pearson's *r*
	Mean run length (conjunction search)	Clarke et al. (2022)	0.88	Pearson's *r*
				
Split-Half Line Segment (SHLS)	Accuracy (hard targets)	Clarke et al. (2022)	[0.71, 0.89]^†^	Pearson's *r*
				
Value driven attentional-capture	RT cost	Anderson & Kim (2019)	0.11	Pearson's *r*
	% Trials with initial fixation on high-value distractor	Anderson & Kim (2019)	0.80	Pearson's *r*
				
Dot-probe task	RT cost	Staugaard (2009)	0.20^‡^	Pearson's *r*
		Schmukle (2005)	0.09	Pearson's *r*
	% Trials with initial fixation on fear face	Price, Kuckertz, Siegle, Ladouceur, Silk Ryan, & Amier, et al. (2015)	0.71	ICC
				
Attentional blink	Switch AB	Dale & Arnell (2013)	0.62	Pearson's *r*
		Dale & Arnell (2013)	0.39	Pearson's *r*
				
Change detection task	K/capacity	Dai et al. (2019)	0.70	Pearson's *r*
				
Visuospatial N-back task	Mean accuracy 2-back	van Leeuwen et al. (2007)	0.16	Pearson's *r*
		Hockey & Geffen (2004)	0.54	Pearson's *r*
	Mean accuracy 3-back	van Leeuwen et al. (2007)	0.70	Pearson's *r*
		Hockey & Geffen (2004)	0.73	Pearson's *r*
Visuoverbal N-back task	Mean accuracy 3-back	Soveri et al. (2018)	0.57	ICC

*Note*. *Clarke et al. (2022) re-analyzed data from Hartkamp and Thornton (2017) to estimate test-retest reliability for the foraging paradigm. ^†^The 95% confidence interval for Pearson's correlation coefficient. ^‡^Reliability for 500 ms angry condition reported.

## Discussion

Assessing test-retest reliability of a behavioral paradigm is essential if we wish to use the task to explore individual differences, but this is still not common practice in vision science. In the current paper, we propose appropriate methods to evaluate reliability of attention and perception tasks, and we used these methods to determine the test-retest reliability of four commonly used tasks: MoCo, UFOV, MOT, and change detection. We also reviewed the existing reliability metrics of vision science tasks within the wider literature to date and present the reported reliability statistics on a comprehensive variety of tasks. Our own data, as well as the additional data reviewed, reveal that there are a wide range of reliability scores for tasks commonly used in vision science, with some as high as 0.91 (e.g., RT cost on the Eriksen flanker task, Wöstmann, Aichert, Costa, Rubia, Möller, & Ettinger, 2013) and others as low as 0 (e.g. global RT cost on the Navon task, Hedge et al., 2018b). We propose that the test-retest reliability of a given task, or more specifically within the particular measure and specifications used, should be considered before attempting to use the task to investigate individual differences in performance.

Certain measures we tested exceeded good (near excellent) standards of reliability, particularly VWM capacity (ICC = 0.77) and the most difficult (i.e, furthest peripheral) measures of the UFOV task (ICC = 0.74). As in Hedge et al. (2018b), these high ICCs were accompanied by the highest levels of between-participant variance, demonstrating that there must be sufficient performance variation between individuals to allow for high test-retest reliability. Essentially, if we wish to explore how performance on a vision task corresponds to differences in another trait, there must also be substantial differences in performance on the task itself. Our results are also in line with another recent paper (Dai et al., 2019) that focused on the test-reliability of a standard VWM task (e.g., Luck & Vogel, 1997), suggesting that the measures of test-reliability themselves may indeed be quite consistent. However, it must be noted that other work has revealed striking differences between reliability scores for the same task; for instance, van Leeuwen, van den Berg, Hoekstra, and Boomsma (2007) found rather low test-retest reliability for the error cost on the Eriksen flanker task (*r* = 0.29 and *r* = 0.14) whereas others have found higher reliability for the same (ICC = 0.65, Wöstmann et al., 2013; ICC = 0.72, Hedge et al., 2018b). These discrepancies could be partly explained by differences in participants’ performance in these studies: for example, participants in van Leeuwen et al.'s (2007) study reached ceiling for both congruent and incongruent conditions, resulting in a very small error cost rate (*M* = 0.08% and 0.02%) and little between-participant variance. In comparison, participants in Hedge et al.'s (2018b) study showed lower accuracy scores in the incongruent condition relative to the congruent condition, resulting in a much higher error cost rate (*M* = 8.95%) and more variation between participants.

Some discrepancies in reliability scores may also be accounted for by variation in the exact parameters selected for the tasks; the exact specifications of a given task may vary wildly (e.g. Jones, Worrall, Rudin, Duckworth, & Christiansen, 2021; Parsons, Kruijt, & Fox, 2019). There are also often numerous measures within a task and/or different indices that may be used for performance assessment. Test-retest reliability appears to increase as task difficulty increases; for example, for the UFOV task, we assessed test-retest reliability for performance when accurately identifying the location of the probe when it appeared in the innermost, middle, and outermost locations (relative to central fixation). As expected, accuracy declined as distance from fixation increased, but ICCs increased as distance from fixation increased. There were also significant practice effects for all four of the UFOV measures; it is possible that this contributed to the relatively poor reliability of the easier condition in this task (inner ring accuracy), as participants were close to ceiling in session 2. The distinction between *task* and *measure* is important when considering suitability for assessing individual differences, as certain measures within the same task may be far more reliable than others.

If accuracy performance is near ceiling for the easier measures, it is unlikely that there is substantial intraparticipant variability to allow for the space to see consistent and reliable differences in performance between individuals. A similar pattern of results is shown by Dai and colleagues (2019) who observed a rising trend in the test-retest reliability coefficients as the memory set size increased: Pearson's *r*s of 0.50, 0.57, 0.65, and 0.76 were found for set sizes three, four, five, and six, respectively. Soveri, Lehtonen, Karlsson, Lukasik, Antfolk, and Laine (2018), who investigated the test-retest reliability of frequently used executive tasks in healthy adults, also demonstrate a similar trend in results. Among a battery of executive tasks was a visuoverbal N-back working memory task; in this task, numbers one to nine were presented, and participants indicated whether this number matched the number either in the previous trial (1-back condition) or the number three trials back (3-back condition). As difficulty increased (i.e. the load factor increased), RT to respond also increased, as expected. The ICC values for the RT measure increased with increasing difficulty with ICC values of 0.48 and 0.73 for the one-back and three-back conditions, respectively. These findings, together with our results, suggest that task measures which are objectively more difficult may be more reliable. Using a more difficult task measure can help optimize between-participant variation, a core component allowing appropriate exploration of test-retest reliability.

The importance of considering the particular measures and parameters used is also apparent when interpreting our reliability results for our MOT task. We compared performance on the maximum items an individual could track as well as the threshold for the number of items retained in VWM, and the ICCs were quite low (0.41 and 0.36, respectively). At first glance, these results may suggest MOT is a particularly unreliable task and not well suited for the study of individual differences; however, there is an almost infinite range of parameters that can be employed when testing MOT performance (Meyerhoff, Papenmeier, & Huff, 2017; Scholl, 2009). For example, the calculations to assess performance on the task can look quite different, depending on whether the probe-one or mark-all method is used (Hulleman, 2005). Our version of the MOT task used the mark-all method (i.e. participants were asked to correctly identify all target items), but even within the mark-all method, the task itself can vary wildly according to the speed and the trajectory of the items to be tracked. Additionally, the staircase we used varied the number of items presented, which may have limited the variability in terms of what we could explore. Alternatively, the speed of the items can be titrated rather than the number to provide a finer-grained threshold estimation (e.g. Bowers, Anastasio, Sheldon, O'Connor, Hollis, Howe, & Horowitz, 2013; Meyerhoff, Papenmeier, Jahn, & Huff, 2016). Such a measure may yield significantly more intraparticipant variability and thus may be more suitable for evaluating individual differences (e.g. Meyerhoff & Papenmeier, 2020).

However, it is critical that any increased variance reflects the construct of interest rather than simply any between-participant variance. Behavioral performance in cognitive tasks is multifaceted, and we are nearly always capturing multiple processes. For example, the Stroop effect is commonly assumed to isolate inhibitory ability. By subtracting performance in a congruent condition from an incongruent condition, we assume that we have controlled for general factors like processing speed and strategy. However, recent modeling work has questioned this assumption. Parameters representing processing speed and strategy correlate with reaction time differences in tasks like the Stroop and flanker (Hedge, Powell, & Sumner, 2018a; Hedge, Powell, Bompas, Vivian-Griffiths, & Sumner, 2018c), correlate across tasks (Hedge, Bompas, & Sumner, 2020), and show higher reliability than parameters representing inhibitory processes (Hedge, Vivian-Griffiths, Powell, Bompas, & Sumner, 2019). We might try to increase variability in a reaction time measure by encouraging participants to be accurate and improve reliability as a result, but in doing so we may unwittingly increase the contribution of individual differences in strategic slowing to our measure. Intelligence tends to be highly correlated with performance on perception tasks, and this is largely mediated by individual differences in attentional control (Tsukahara et al., 2020). Many between-participant differences in vision tasks may be also driven by higher-level differences in motivation and cognition rather than core differences in vision.

Like many psychology studies relying on undergraduates as participants, our sample is from a western, educated, industrialized, rich, and democratic (WEIRD) society (Henrich et al., 2010) and consists of overwhelmingly female participants. In line with known gender differences in performance on the MOT task (Skogsberg et al., 2015), we found that men outperformed women in that they had a higher threshold for the number of items correctly tracked. Additionally, men were more accurate for one of the four measures on the UFOV task (accuracy for the outermost location). There were also differences in the variances for men and women on one session for one of the measures (mean number of items tracked for the MOT), but these differences were rare and inconsistent (i.e. not evident in both sessions for the same measure). Importantly, however, the ICCs for men and women on these measures were roughly equivalent. The primary purpose of our analyses is to demonstrate whether a task captures consistent performance at test and retest rather than to characterize the way in which people, as a group, perform. The reliability of these measures in other populations is necessarily an empirical question. Reliability is a function of between-subject variance and measurement error, and these could be different in other populations of interest (e.g. clinical groups). However, our data show that in the case of gender, our reliability estimates are consistent despite differences in performance.

A variety of studies have also assessed the internal reliability of similar measures. Tasks such as MOT demonstrate high levels of internal reliability (e.g. 0.80, Skogsberg et al., 2015; 0.92, Treviño et al., 2021), but high internal reliability of a task does not necessarily translate to high test-retest reliability (i.e. our MOT reliability scores were rather low). We would expect internal reliability to be a constraint on test-retest reliability such that poor internal reliability would likely preclude the possibility of good test-retest reliability (i.e. if a measure cannot measure a construct consistently at one time point, it is unlikely to be able to do so across multiple time points). However, even measures with high internal reliability may not reveal consistent performance across two time points, and the critical purpose of assessing test-retest reliability for the purposes of exploring individual differences is to evaluate the stability of a measure over time.

**Figure 4.**
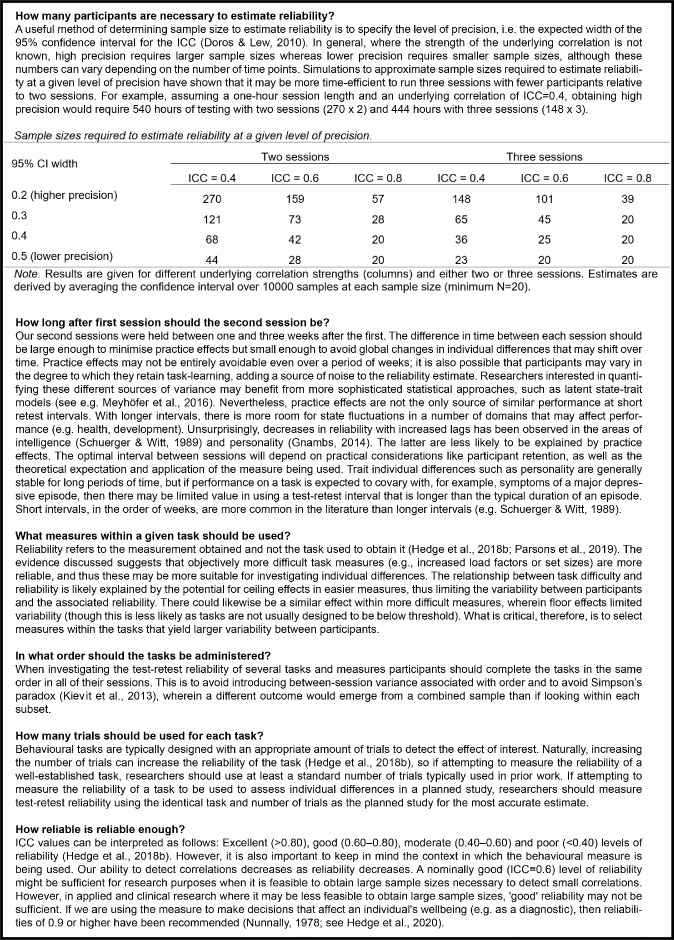
Recommendations for evaluating test-retest reliability (see Doros & Lew, 2010; Gnambs, 2014; Hedge et al., 2018b; Hedge et al., 2020; Kievit et al., 2013; Meyhöfer et al., 2016; Nunnally, 1978; Parsons et al., 2019; Schuerger & Witt, 1989; as referenced above).

Finally, it is important to highlight that a low ICC is not a damning metric for a measure overall. For example, measures within common inhibition tasks, such as the Eriksen flanker task (e.g. RT cost), tend to have low reliability (see Table 4) but demonstrate highly robust experimental effects (Rouder, Kumar, & Haaf, 2019). Such measures are not without merit and can be useful in asking questions of an experimental nature, rather than correlational, such as the underlying cognitive and neural processes involved in human behavior (e.g. White, Ratcliff, & Starns, 2011). Tasks with a low ICC can be just as useful for examining cognitive processes as those with high ICCs; the difference is that they are useful for asking different questions: the low ICCs in such tasks are typically the result of very low between-participant variability, which is an excellent feature when the primary question is within-participant differences between conditions. Test-retest reliability does not speak to the quality of the task in general but rather speaks to the quality of the task for the explicit purpose of investigating individual differences. We recommend that when researchers are aiming to explore individual differences in performance on vision tasks, considering the test-retest reliability is crucial. With this in mind, we propose a useful guide for evaluating test-retest reliability (see Figure 4).

## Conclusion

Many vision science tasks were designed with the intention of minimizing variance between participants in order to assess phenomena common to human visual systems generally. However, these same tasks are now being used to assess how performance varies across a population in accordance with individual differences between participants. Before attempting to use a perceptual task to assess individual differences, researchers should consider its test-retest reliability.

We collected our own data on common vision tasks for which test-retest had not yet been established at the time of data collection and reported on the known test-retest reliabilities for a wide variety of other perceptual tasks. In line with previously established reliabilities, we found a range of reliability scores between both tasks and performance indices within the tasks themselves. As a result of our work and in line with Hedge et al.'s (2018b) work on cognitive control tasks, we provide a useful guide for assessing test-retest reliability of perception and attention tasks, and we advise researchers interested in exploring individual differences to consider this important metric when developing their studies. We argue that this is a necessary step in evaluating whether a particular task, or the particular implementation of the task, is suitable for the exploration of individual differences.

## Supplementary Material

Supplement 1
