# Supplementary material for: Test-retest reliability for common tasks in vision science
Source: J Vis. 2022 Jul 29;22(8):18. doi: 10.1167/jov.22.8.18 (PMC9344221; doi:10.1167/jov.22.8.18)
Supplement: Supplement 1 [file jovi-22-8-18_s001.pdf]

Supplementary Material A

Note. Because of the limited number of male participants, the ICC values for males are not especially precise. The high male ICC for UFOV Inner Accuracy may be anomaly that would not hold true for a larger sample.

Task Measure	Gender (N)	Session 1 M (SD)	Session 2 M (SD)	ICC	Main Effect (Session)	Session x Gender	Main Effect (Gender)	Levene's Session 1	Levene's Session 2
MoCo Threshold	Male (19)	0.27 (0.11)	0.27 (0.13)	0.66	*F*(1, 139) = 0.14, η^2_p = 0.00, *p* = 0.71	*F*(1, 139) = 0.02, η^2_p = 0.00, *p* = 0.88	*F*(1, 139) = 0.51, η^2_p = 0.00, *p* = 0.48	*F*(1, 139) = 0.09, *p* = 0.77	*F*(1, 139) = 2.69, *p* = 0.10
	Female (122)	0.29 (0.11)	0.28 (0.09)	0.59					
UFOV Number Accuracy	Male (19)	0.94 (0.06)	0.96 (0.03)	0.21	*F*(1, 135) = 6.25, η^2_p = 0.04, *p* = 0.01	*F*(1, 135) = 0.24, η^2_p = 0.00, *p* = 0.63	*F*(1, 135) = 0.02, η^2_p = 0.00, *p* = 0.90	*F*(1, 135) = 0.33, *p* = 0.57	*F*(1, 135) = 1.01, *p* = 0.32
	Female (118)	0.94 (0.06)	0.96 (0.05)	0.52					
UFOV Inner Accuracy	Male (19)	0.86 (0.21)	0.95 (0.12)	0.73	*F*(1, 135) = 21.48, η^2_p = 0.14, *p* <.001	*F*(1, 135) = 0.81, η^2_p = 0.01, *p* = 0.37	*F*(1, 135) = 2.61, η^2_p = 0.02, *p* = 0.11,	*F*(1, 135) = 1.09, *p* = 0.30	*F*(1, 135) = 1.41, *p* = 0.24
	Female (118)	0.78 (0.18)	0.91 (0.17)	0.30					
UFOV Middle Accuracy	Male (19)	0.26 (0.18)	0.32 (0.21)	0.75	*F*(1, 135) = 10.65, η^2_p = 0.07, *p* = 0.001	*F*(1, 135) = 0.001, η^2_p = 0.00, *p* = 0.97	*F*(1, 135) = 0.36, η^2_p = 0.00, *p* = 0.55	*F*(1, 135) = 0.07, *p* = 0.80	*F*(1, 135) = 0.01, *p* = 0.95
	Female (118)	0.23 (0.17)	0.30 (0.20)	0.58					
UFOV Outer Accuracy	Male (19)	0.18 (0.13)	0.24 (0.18)	0.69	*F*(1, 135) = 9.54, η^2_p = 0.07, *p* = 0.002	*F*(1, 135) = 3.44, η^2_p = 0.03, *p* = 0.07	*F*(1, 135) = 7.29, η^2_p = 0.05, *p* = 0.01	*F*(1, 135) = 0.65, *p* = 0.42	*F*(1, 135) = 3.16, *p* = 0.08
	Female (118)	0.12 (0.12)	0.14 (0.14)	0.75					
MOT Max Items	Male (21)	6.14 (0.65)	6.14 (0.73)	0.39	*F*(1, 140) = 0.002, η^2_p = 0.00, *p* = 0.96	*F*(1, 140) = 0.002, η^2_p = 0.00, *p* = 0.96	*F*(1, 140) = 3.84, η^2_p = 0.03, *p* = 0.05	*F*(1, 140) = 0.25, *p* = 0.62	*F*(1, 140) = 2.67, *p* = 0.11
	Female (121)	5.89 (0.66)	5.90 (0.60)	0.40					
MOT Threshold	Male (21)	4.88 (0.59)	4.79 (1.03)	0.25	*F*(1, 140) = 0.21, η^2_p = 0.00, *p* = 0.65	*F*(1, 140) = 0.25, η^2_p = 0.00, *p* = 0.62	*F*(1, 140) = 8.98, η^2_p = 0.06, *p* = 0.003	*F*(1, 140) = 1.94, *p* = 0.17	*F*(1 ,140) = 6.22, *p* = 0.014
	Female (121)	4.41 (0.66)	4.47 (0.74)	0.35					
VWM Capacity (k)	Male (19)	2.44 (0.83)	2.34 (1.00)	0.80	*F*(1, 135) = 0.04, η^2_p = 0.00, *p* = 0.85	*F*(1, 135) = 1.70, η^2_p = 0.01, *p* = 0.19	*F*(1, 135) = 0.02, η^2_p = 0.00, *p* = 0.88	*F*(1, 135) = 0.83, *p* = 0.36	*F*(1, 135) = 1.65, *p* = 0.20
	Female (118)	2.33 (0.75)	2.40 (0.80)	0.77					
